# United against cancer: prevention to end-of-life care—highlights from the Uganda Cancer Institute–Palliative Care Association of Uganda Joint International Conference on Cancer and Palliative Care and the 7th Palliative Care Conference, 24–25 August 2017, Kampala, Uganda

**DOI:** 10.3332/ecancer.2017.790

**Published:** 2017-12-14

**Authors:** Julia Downing, Henry Ddungu, Fatia Kiyange, Mwazi Batuli, James Kafeero, Harriet Kebirungi, Rose Kiwanuka, Noleb Mugisha, Eddie Mwebesa, Mark Mwesiga, Elizabeth Namukwaya, Nixon Niyonzima, Warren Phipps, Jackson Orem

**Affiliations:** 1 Makerere University, International Children’s Palliative Care Network, Uganda; 2 Palliative Care Association of Uganda, Kampala, Uganda; 3 Uganda Cancer Institute, Kampala, Uganda; 4 African Palliative Care Association, Kampala, Uganda; 5 Makerere/Mulago Palliative Care Unit, Kampala, Uganda; 6 Hospice Africa Uganda, Kampala, Uganda

**Keywords:** cancer care, palliative care, Uganda, policy, integration, education, research, paediatrics, East Africa

## Abstract

The Uganda Cancer Institute (UCI) and the Palliative Care Association of Uganda (PCAU) jointly hosted an international conference on cancer and palliative care in August 2017 in Kampala, Uganda. At the heart of the conference rested a common commitment to see patient care improved across Uganda and the region. The theme – United Against Cancer: Prevention to End-of-Life Care – reflected this joint vision and the drive to remember that cancer care should include prevention, early diagnosis and screening, treatment, rehabilitation and palliative care. The conference brought together 451 delegates from 17 countries. The key themes of the conference included: the importance of the World Health Assembly Resolutions on Palliative Care (2014) and cancer care (2017); the need to develop a National Cancer Control Programme; strategies for effective cancer diagnosis and treatment in low- and middle-income countries; advocacy, human rights and access to essential medicines, including access to opioids and nurse prescribing; paediatric care; leadership and commitment; collaboration; resources (financial and human), the recognition that palliative care is not limited to cancer care and the importance of learning from each other. The conference also gave the opportunity to celebrate the 50th Anniversary of the UCI, with a celebration dinner attended by the Minister of Health and the US Ambassador. Participants reported that the conference was a forum that updated them in all aspects of cancer and palliative care, which challenged their knowledge, and was enlightening in terms of current treatment options for individuals with cancer. The benefits of having a joint conference were recognised, allowing for further networking between cancer and palliative care organisations. This conference, highlighting many developments in cancer and palliative care, served as a unique opportunity to bring people together and unite them in developing cancer and palliative care.

## Introduction

Having a National Cancer Control Programme (NCCP) is important as it ‘*offers the most rational means of achieving a substantial degree of cancer control, even where resources are severely limited*’ [1]. Thus, the World Health Organization (WHO) recommends the development of such a programme ‘*wherever the burden of disease is significant, there is a rising trend of cancer risk factors and there is a need to make the most efficient use of limited resources*’ [1]. NCCPs have five key elements: (1) prevention, (2) early detection and screening, (3) diagnosis and treatment, (4) palliative care and (5) policy and advocacy [2]. This joint Uganda Cancer Institute (UCI) and Palliative Care Association of Uganda (PCAU) conference, United Against Cancer: Prevention to End-of-Life Care, covered all aspects of a cancer control programme, with issues around the challenges in prevention and early detection and screening in Uganda discussed, and advances in diagnosis and treatment, developments in palliative care and issues around policy and advocacy for cancer and palliative care highlighted.

The conference was also the 7th Ugandan Palliative Care Conference. Palliative care is ‘*an approach that improves the quality of life of patients and their families facing the problem associated with life-threatening illness, through the prevention and relief of suffering by means of early identification and impeccable assessment and treatment of pain and other problems, physical, psychosocial and spiritual*’ [3]. At the heart of this joint UCI–PCAU conference was the commitment to improve the provision of cancer care and palliative care within Uganda and Eastern Africa, with a desire to share and learn from each other.

It is an exciting time to be working in the fields of cancer and palliative care. The conference marked the 50th anniversary celebrations of the UCI providing cancer care and research. UCI is a regional centre of excellence and has provided care to individuals within Uganda, Western Kenya, the Democratic Republic of Congo, Rwanda and Burundi, amongst others. They have also conducted some of the ground-breaking research on cancer in Africa and globally. UCI is a public medical care facility under the Ministry of Health of Uganda, with a focus on research, training, consultation, prevention and cancer treatment in areas of paediatrics, oncology, gynaecology, radiotherapy, surgery and palliative care. It has an inpatient capacity of 80 beds, with an average of 4,000 new cancer patients and over 40,000 patient visits annually. It is affiliated with the Mulago Hospital complex and the Makerere University College of Health Sciences [4].

PCAU is a member organisation for palliative care professionals, care providers and organisations in Uganda, established in 1999. Their mission is to promote and support affordable and culturally appropriate palliative care throughout Uganda via strengthening the healthcare system in Uganda. Its focus areas include: integrating palliative care services in every district through training, mentorship and support supervision; increasing awareness and understanding of palliative care; and to be a central hub for data and information by collecting, storing and disseminating information to improve services. PCAU works through the national framework for palliative care and is mandated by the Ministry of Health to provide leadership and coordination for the scale-up of palliative care services in Uganda [5].

## Conference background

Cancer is one of the leading causes of morbidity globally and an important public health concern, with the number of new cases annually expected to increase from 14.1 million in 2012 to 21.6 million by 2030 [6]. Uganda has high morbidity and mortality due to cancer, with the burden of cancer rising at an alarming rate due to environmental agents, lifestyle, infection and the HIV epidemic [7]. In 2015, it was identified that there were more than 200,000 cases of cancer in the country with about 46,970 cancer-related deaths, set to rise to over 300,000 over the next five years [8]. Alongside this, 80% of individuals with cancer in Uganda either report or are diagnosed late, making cure an option that is still far from reality and the need for palliative care an important priority [9].

Uganda is seen as one of the leading countries in sub-Saharan Africa for the provision of palliative care [10]. In the recently published Atlas of Palliative Care in Africa [11], it was noted that there are 216 hospitals providing hospital-based palliative care services (20% of hospitals) and 13 stand-alone hospices, along with home-based care services, thus palliative care is integrated into the healthcare system, though some services are a lot stronger than others, and there is a need for more children’s palliative care services [12] alongside those already established such as at Mildmay Uganda [13]. The Ministry of Health pays for morphine for all palliative care patients, with national guidelines having been developed. Education and training on palliative care is also well developed, with palliative care being integrated into some undergraduate and postgraduate training, particularly in the medical school at Makerere University. There is also a diploma/degree in palliative care affiliated to Makerere University with a Master’s programme due to start soon [14, 15], and a Diploma in Paediatric Palliative Care run by Mildmay Uganda [16] alongside an award-winning nurse leadership fellowship programme [17].

In the past few years, there have been key developments in cancer and palliative care with the World Health Assembly (WHA) passing resolutions that are critical in guiding us into the future for cancer and palliative care service provision. In May 2017, the WHA passed a resolution on cancer prevention and control within the context of an integrated approach for cancer care [6]. Within this resolution, member states are urged to scale up NCCPs as part of the national response to non-communicable diseases. This is in line with the 2030 Agenda for Sustainable Development [18] and the sustainable development goals, in particular that of Goal 3: good health and well-being [19]. In 2014, the WHA also passed a resolution on the strengthening of palliative care as a component of comprehensive care throughout the life course [20]. Key to the development of palliative care is integration, with member states being encouraged to integrate palliative care across the continuum of care, for all age groups, and all diseases. The implementation of these resolutions will enable each of us to work towards realising our goals in developing cancer and palliative care.

These themes were highlighted in the opening plenary of the conference, where Dr Emmanuel Luyirika [African Palliative Care Association (APCA)], Dr Cherian Verghese (WHO) and Prof Charles Olweny (UCI) noted the importance of the WHA resolution on cancer and palliative care. Together they highlighted some of the areas where we still need to focus our energies, for example: the limited access to radiotherapy in many countries across Africa, including Uganda [21–23]; the lack of adequate cancer registries and NCCPs; the challenge that many families of cancer patients face bankruptcy due to the cost of treatment as well as all of the out-of-pocket expenses incurred; the need to balance the different areas of a cancer control programme, such as early detection, but if we are detecting early, we then need to be able to diagnose and treat; and extending nurse prescribing for palliative care to increase access across the region. However, they also recognised the tremendous progress that has been seen in Uganda in both cancer and palliative care. The contribution of Dr Burkiitt to cancer care across the region was acknowledged, along with the seminal work of the UCI over the years, both in terms of the provision of care and the frontline research into cancer care. The role and commitment of the Ministry of Health was acknowledged, with Uganda being seen as a leader in cancer and palliative care within the region, which could not have happened without the support of the government. Recommendations included: the implementation of the WHA resolutions on cancer and palliative care; the need to increase the number of radiotherapy machines in the region; development of NCCPs in all countries; a review of financing for cancer and palliative care with a rollout of health insurance coverage; focus on prevention of prostate cancer, Kaposi’s sarcoma and breast cancer; the need to think beyond medicine to ensure care is holistic and includes other aspects such as nutrition; and the need for ongoing political commitment to increase access to cancer and palliative care throughout Uganda. Such a political will was echoed by the State Minister of Health for General Duties, the Hon Sarah Opendi and the Prime Minister of the Republic of Uganda, the Rt Hon Dr Ruhakana Rugunda, who officially opened the conference. In his opening remarks Dr Rugunda noted that ‘*Through solidarity we should be able to reduce the power of cancer …and… Collaborations and partnerships are key in implementing successful programmes based on the WHA resolutions on cancer and palliative care’*. Thus, setting the scene for the rest of the conference as we stand ‘*United Against Cancer*’.

## Conference summary

The conference, held at the Speke Resort Munyonyo in Kampala, brought together 451 delegates from 17 countries from around the world. Of these, 12 were African countries, with delegates from Botswana, Burundi, Ethiopia, Ghana, Kenya, Malawi, Nigeria, Rwanda, Tanzania, Uganda, Zambia and Zimbabwe. Other countries represented included: Canada, Ireland, the Ukraine, the United Kingdom and the United States. The conference brought together clinicians, academics, human rights advocates, lawyers, clergy, researchers, social workers, policy makers, Ministry of Health officials, donors and members of the press, representing over a hundred organisations to share lessons and adopt the best practices for cancer and palliative care. Twenty national, regional and international organisations had exhibition stands, the media were in abundance and interpreters translated the conference into sign language. The conference was organised into seven tracks: (1) capacity building, (2) service provision, (3) advocacy, (4) research/monitoring and evaluation, (5) working with children, (6) human rights and legal issues and (7) psychosocial and spiritual issues. The scientific programme included a variety of plenary sessions (14 papers plus country updates), 38 oral breakout presentations, four workshops, one side event on access to controlled medicines and 93 poster presentations over the two days. Presentations were given across the continuum of care, from prevention through to end-of-life care, across the age span, and whilst the main focus was on cancer care, the provision of palliative care for other conditions was also addressed.

During the dinner, which celebrated the 50th anniversary of the UCI, a pictorial presentation on the history of UCI was presented, which linked in to an exhibition being held in the Afri-Art Gallery entitled ‘Staying Alive’. Dr Jackson Orem, Executive Director, UCI, presented the achievements and future plans for UCI, and remarks were provided by Dr Bruce Clurman, Deputy Director and Executive Vice President of Fred Hutchinson Cancer Research Center in the USA, the US Ambassador, Her Excellency Deborah Malac and the Guest of Honour, the Hon Dr Jane Ruth Aceng, the Minister of Health for the Republic of Uganda gave a speech and presented some awards. Awards were given by the UCI in recognition of individuals and organisations’ contribution to the UCI and cancer care in Uganda. The PCAU also gave three awards, two of which were in recognition of individuals’ contributions to the founding of PCAU and the development of palliative care in Uganda, and were presented to Fatia Kiyange and Prof Dr Julia Downing. The morphine production unit at Hospice Africa Uganda was also given an award to recognise the important role they had played, along with the challenges of setting up the unit in the first place. The Celebration dinner was rounded off by music from the Afrigo Band accompanied by dancing.

## Key conference themes

The key themes identified from the conference were as follows:

- The importance of the WHA Resolutions on Palliative Care [20] and cancer care [6]
- The need to develop an NCCP in Uganda
- Strategies for effective cancer diagnosis and treatment in low- and middle-income countries (LMICs)
- Advocacy, human rights and access to essential medicines, including nurse prescribing
- Paediatric care
- Leadership and commitment
- Collaboration
- Resources (financial and human)
- The recognition that palliative care is not limited to cancer care.

Alongside these, there were many cross-cutting issues identified across the conference such as the need for research and data, the need for policy, education and so on.

### The importance of the WHA resolutions on palliative care and cancer care

Throughout the conference, the importance of these two resolutions from the WHA was emphasised. It was noted that the WHA resolution on palliative care [20] was the first time that the issue of palliative care had been discussed at the WHA, and its importance was recognised through the adoption of this resolution. Since then the resolution has been used as an advocacy tool throughout the region to encourage member states to implement the resolution, ensuring that palliative care is ‘integrated’ within the health system. Emphasis has been put on the WHO building blocks for health systems strengthening [24], with the development of palliative care being recognised as a health systems strengthening approach, with examples being seen in various programmes, including the ‘*Integrate programme*’ in Uganda, Zambia, Kenya and Malawi [25]. Alongside this, there is a growing recognition of the need for Universal Health Coverage (UHC) – with palliative care being seen as an integral part of UHC. Indeed, the WHO states that UHC means ‘*that all people and communities can use the promotive, preventive, curative, rehabilitative and palliative health services they need, of sufficient quality to be effective, while also ensuring that the use of these services does not expose the user to financial hardship*’ [26]. It has therefore become a major goal and priority for WHO.

Likewise, for cancer care, whilst the resolution was only passed three months ago, its importance is being recognised amongst advocates. It calls for the development, implementation and monitoring of programmes at all levels of cancer control, i.e. prevention, early detection and screening, diagnosis, treatment and palliative care and across all age groups, highlighting the need for cancer treatment for children [6]. The adoption of the resolution by the WHA recognises the need for ongoing developments within the field of cancer care, alongside that of non-communicable diseases. Since the previous cancer resolution in 2005 [27], more has become known on the management of the disease and there has been a recognition of the impact of non-communicable diseases on mortality and morbidity with an increase in life-limiting NCDs [28], with Africa seeing the highest rate of increase of deaths from NCDs [29]. Thus, together, this emphasis on NCDs and the resolution on cancer care can be highlighted in our ongoing advocacy to governments and other organisations to support the development of cancer care across the continuum.

Many of the presentations throughout the conference highlighted either one or both of these resolutions, alongside the management of NCDs, the sustainable development goals and UHC. Thus, showing that whilst striving to improve the quality of care for the individual patient within Uganda, service providers are also aware of the political climate in which they are working, and the importance of utilising such information for gaining governmental support. Throughout the conference, the need for governmental support was recognised, and the support provided was acknowledged, whilst stressing the need for ongoing support for both cancer and palliative care service development, education and research.

### The need to develop an NCCP in Uganda

The importance of having an NCCP in Uganda was highlighted from the start of the conference, in order to guide a comprehensive and integrated approach to care, and was a recurring theme seen throughout many of the presentations. The WHA cancer resolution urges member states ‘*to develop, as appropriate, and implement national cancer control plans that are inclusive of all age groups; that have adequate resources, monitoring and accountability; and that seek synergies and cost-efficiencies with other health interventions*’ [6]. Without a clear NCCP that has been well structured and thought out, covers the spectrum of planning, prevention, early detection and screening, diagnosis and treatment, palliative care and advocacy and policy, countries will not move forward in the provision of an integrated approach to cancer care. Currently, it was noted that prevention and secondary prevention is being overshadowed by treatment. The WHO guidelines on how to develop and implement an effective cancer control plan was a response to the initial WHA cancer resolution in 2005, and sets out practical advice on how to set up a cancer control programme, particularly in LMICs such as Uganda, and can be used alongside the policies and managerial guidelines for NCCPs [2].

The development of an integrated and sustainable cancer control strategy and programme within Uganda is underway, with the key stakeholders and government involved in its development. Whilst recognising the challenges in doing this, and in implementing such a programme, presenters and delegates alike are united in the belief that this will help planning and ensuring that there is emphasis given to all stages of the NCCP, such that eventually less patients will present late, and more will present earlier on in the disease process whilst cure is still a possibility, thus having an impact on the treatment being provided.

### Strategies for effective cancer diagnosis and treatment in LMICs

It was recognised that LMICs share the biggest proportion of cancer burden of disease due to the lack of access to appropriate technologies and systems and services. Whilst one-third of global cancer care is needed in sub-Saharan Africa, there is a shortage of pathology and treatment options here, with the issue of limited resources cutting across all LMICs within the region. It was stressed that it is important to advocate for improvement in services across the cancer continuum, ranging from encouraging early screening, e.g., for cervical cancer, improved cancer diagnosis and early access to palliative care services. A range of options was discussed for scaling up treatment for different cancers, such as cervical cancer, prostate cancer and childhood cancers, the importance of understanding the biology of cancer and genetics, precision medicine for individual tailoring of treatment according to individual patient pathology, access to treatment, such as radiotherapy, blood transfusions, medications and so on, and ensuring that treatment is available for all including vulnerable populations such as children, the disabled, the elderly and refugees or internally displaced individuals.

The outcomes of care are important and there were a variety of presentations that demonstrated improvements in the outcomes of cancer treatment, such as a 94% overall survival rate for Burkitt’s lymphoma, improved outcome for cervical cancer, and improvements in outcomes of care through the provision of palliative care at the Mulago National Referral Hospital, to name but a few. Throughout, it was stressed that the care we give is not just about physical care, but we need to consider psychological, social and spiritual aspects of care as well, and this can be done in a variety of ways, including building compassionate communities. Dr Anne Merriman stressed the importance of not losing sight of the individuals we are caring for and their families, and ensuring that compassion and care are central to all that we do.

Education and the building capacity for cancer and palliative care service provision were seen as key to improve services in LMICs. For many, access to appropriate education can be a challenge, and lessons were shared about a variety of different education programmes such as the Diploma/Degree programme run by the Institutes of Hospice and Palliative Care in Africa (IHPCA) accredited by Makerere University, different ways of building capacity for cancer nursing, continuing education in palliative care and so on. Alongside capacity building, issues of policy, advocacy and research were raised, with an ongoing need for advocacy for cancer and palliative care alongside service provision, if we are to continually improve the services that we can provide in Uganda and other LMICs. The challenge of undertaking research in LMICs was discussed, along with some of the ethical issues involved, and providing correct support when undertaking research to ensure that participants do not just say what they think the researchers want to hear. National and government recognition of international days was also seen as important, such as World Cancer Day, World Hospice and Palliative Care Day and World AIDS Day. Whilst much of the focus of the conference was understandably on Uganda, snapshots were shared from other countries within the region such as Kenya, Rwanda, Tanzania and Zambia.

### Advocacy, human rights and access to essential medicines, including nurse prescribing

An ongoing issue for both cancer and palliative care is access to essential medicines, including controlled medicines such as oral morphine. The global palliative care organisations – the International Association of Hospice and Palliative Care (IAHPC), the International Children’s Palliative Care Network (ICPCN) and the Worldwide Hospice and Palliative Care Alliance (WHPCA) have an ongoing engagement drive with the UN organisations and WHO to position and include the palliative care agenda in each of their agendas, e.g., the United Nations General Assembly SS, (UNGASS) the African Union (AU), the WHA, the Commission on Narcotic Drugs, the Human Rights Council, the special rapporteurs sessions and so on. Cancer and palliative care have been highlighted through the recent cancer [6] and palliative care resolutions [20], but also through Agenda 2030 [18] and the Sustainable Development Goals within Goal 3 [19] and UHC [26]. Therefore, there is leverage for ongoing advocacy in these areas.

Accessibility and availability of medicines, in particular controlled medicines such as oral morphine, is an ongoing challenge within palliative care and it is important that a balance is struck between accessibility and availability alongside the misuse of drugs. Issues around this were discussed not only in the main conference, but also in the side event on accessibility to medicines, which was convened by the PCAU in partnership with the Ugandan Ministry of Health and supported by the Open Society Institute for Eastern Africa. The side event, attended by 44 invited stakeholders, discussed accessibility and availability of controlled medicines in Uganda, the regulatory framework for controlled medicines and the formation of an ad hoc committee to follow up on issues around nurse and clinical officer prescribing within palliative care.

Whilst Uganda is recognised as the first country to allow nurse prescribing of oral morphine by trained palliative care nurses and clinical officers [30], no full-scale evaluation of nurse prescribing had been conducted until now. The results of an evaluation into nurse prescribing within palliative care, which had been undertaken as part of the nurse leadership programme [17], were shared, demonstrating that nurses can prescribe safely and effectively managing individuals’ pain. Nurse prescribing within the context of palliative care can thus improve accessibility to palliative care and they can do it well. Nurses were encouraged to continue in this role whilst ongoing work continues with the Ministry of Health, the Ministry of Internal Affairs, and the Ugandan Nursing and Midwifery Council, to ensure that the legal mandate that allows them to do this is continued.

The importance of the provision of health care as a universal and human right was stressed while advocating for improvements in cancer and palliative care. Examples were shared from the Uganda Network on Law Ethics and HIV/AIDS (UGANET), of how legal people have a vital role to play in supporting cancer and palliative care provision and two patients gave testimonies about the support. The Uganda Human Rights Commission (UHRC) shared that they had initiated monitoring issues around the right to palliative care in Uganda. The Initiative for Social and Economic Rights (ISER) also empathised that access to treatment is a human right, and they stressed the importance of patients knowing and understanding their rights whilst accessing care, along with the importance of a human rights-based approach to palliative care. In Uganda, PCAU, UGANET, APCA and palliative care service providing sites have successfully piloted a programme on access to justice, which has seen the integration of legal support within palliative care services. Through this initiative, palliative care patients who are unable to pay for legal services are accessing services free of charge.

### Paediatric care

Treatment of childhood cancers and the provision of palliative care for children were integrated throughout the programme. The need is to ensure that any comprehensive paediatric cancer centre has a focus beyond that of the disease, and cares for the child and its family in a holistic manner, providing compassionate care that nurtures hope whilst being realistic. Work has been ongoing at the UCI with regard to phenotype and outcomes of paediatric malignancies and results to date were shared along with a discussion of whether children die of their cancer or the effects of cancer treatments and infections. It was encouraging to note that whilst survival rates for childhood cancers are still relatively poor (apart from for Burkitt’s lymphoma), they are improving and improvements in diagnosis and treatment methods have helped.

The importance of children having a voice throughout their treatment was stressed, along with the importance of good psychological care. It was recommended that mental health should be incorporated into cancer care for children and adolescents, as the prevalence of depression in children aged 7–17 years with cancer was highlighted as 28%. Thus, the importance of a multidisciplinary team was recognised along with different options to help reduce stressors, including financial for the child and their families, such as the use of hostels whilst receiving treatment, and the role of oncology nurse managers in caring for the children and ensuring that they do not default from treatment. Decision making was also discussed, and how best decisions can be made throughout the disease trajectory, with the importance of open and honest communication, and involving the child as appropriate. Other issues raised included that of pain control for children, the importance of good end-of-life care and the importance of providing grief and bereavement support.

### Leadership and commitment

Good leadership and commitment from the government as well as stakeholders, for cancer and palliative care was stressed throughout the conference. Time and again, it was noted that without this, further strides in development will be limited. Ensuring effective leadership was highlighted along with how best we can build capacity for effective leadership and ensure that we are creating change agents for good governance and political commitment through team-based approaches.

### Collaboration

Creating linkages, networking and collaboration were key recommendations from the conference. Whilst discussions ensued as to how best we could do this, and with whom, e.g., the deaf and disabled, non-cancer and non-palliative care stakeholders and so on, the conference itself was seen as a good opportunity for creating such collaborations. As the first conference to bring together the cancer and palliative care communities within Uganda and Eastern Africa, it offered a platform for sharing, learning from each other and beginning to build networks and discussion opportunities for collaboration which will be ongoing beyond the conference itself. It was recognised that collaboration can enhance service provision, with key issues discussed including relationship building, good communication, truth telling and transparency, and exchanging views and information. Throughout the conference, a range of collaborations and partnerships were discussed, including South-to-South Partnerships, South to North Partnerships, Cancer and Non-cancer partnerships, Palliative care and Cancer partnerships and so on. Collaboration across the cancer centres within Eastern Africa along with the rest of Africa was also strongly recommended. Whilst acknowledging the importance of such collaborations, some of the challenges were also discussed, along with ways to overcome such challenges. However, throughout the conference it was acknowledged that the benefits outweigh the challenges and the partnerships at both the global and local levels are essential.

### Resources (financial and human)

A recurrent theme throughout the presentations and the provision of cancer and palliative care in LMICs is that of resources – not just financial resources but also human resources, lack of equipment, e.g., radiotherapy, to name but a few. Human resources are a key challenge, with a lack of trained people and a lack of access to appropriate training locally, and ensuring that as new techniques are introduced, appropriate capacity is built within Uganda. In order to treat and prevent cancer, we need a stronger human resource of well-trained and skill personnel in this area. Human resources for health are key, along with multidisciplinary teamwork, respect, leadership and so on. In the closing plenary, Prof Francis Omaswa emphasised the need to make a deliberate effort to put in place competent human resources for cancer and palliative care and address the issue of the retention of health workers in Uganda.

Financial challenges were seen as cross-cutting, ranging from the cost of treatment, the impact of illness on finances and the high number of patients and families falling into debt, along with the costs of providing care at the national level and how Uganda can implement UHC within its financial resources.

### The recognition that palliative care is not limited to cancer care

Finally, as the focus of the conference was on both cancer care and palliative care, it was recognised that the majority of the presentations focused on different aspects of the continuum of cancer care, yet palliative care reaches far beyond cancer, with all children and adults with life-limiting and life-threatening conditions needing access to palliative care services. A few presentations focused on this aspect of palliative care such as palliative care for individuals with heart failure, those with renal failure and those with HIV/AIDS. However, it was acknowledged that this needs to be strengthened in future conferences.

## Conclusion

The past few years have seen significant developments in both cancer and palliative care in Uganda, the surrounding region and other LMICs. Emphasis on advocacy, service development, integration, UHC and health systems strengthening at the global, regional and national levels have contributed towards this, along with the support of government and other stakeholders, and the commitment demonstrated by those working in cancer and palliative care. The success of this first combined UCI/PCAU conference was evident, and the celebration of all that has been achieved by the UCI in the past 50 years, along with that within PCAU was evident. Collaboration, leadership and human resources are the key in the ongoing development in these fields, and this conference was a great opportunity for the strengthening of existing collaborations and the development of new ones. As Uganda continues to move forward in the implementation of both the palliative care [20] and cancer care WHA resolutions [6], the development and implementation of the NCCP will have a significant contribution, such that care is provided along the continuum of care for adults and children from screening through to diagnosis, treatment and palliative care, and for all those in need of palliative care, regardless of diagnosis, age or where in Uganda they live.

## Conflicts of interest

The authors declare that they have no conflicts of interest.
